# The Effect of Psychological Distance on Children’s Reasoning about Future Preferences

**DOI:** 10.1371/journal.pone.0164382

**Published:** 2016-10-14

**Authors:** Wendy S. C. Lee, Cristina M. Atance

**Affiliations:** School of Psychology, University of Ottawa, Ottawa, Ontario, Canada; Western University, CANADA

## Abstract

Young preschool-aged children often have difficulty thinking about the future, but tend to reason better about another person’s future than their own. This benefit may reflect psychological distance from one’s own emotions, beliefs, and states that may bias thinking. In adults, reasoning for others who are more socially distant (i.e., dissimilar, unfamiliar other) is associated with wiser and more adaptive reasoning. The current studies examined whether this effect of social distance could be demonstrated in young children’s future thinking. In a future preferences task, 3- and 4-year-olds were shown 5 pairs of child and adult items and selected which ones they would prefer when grown-up. Children answered for themselves, a socially close peer, or a socially distant peer. Social distance was manipulated by varying similarity in Study 1 and familiarity in Study 2. In Study 1, reasoning for similar and dissimilar peers was significantly more accurate than reasoning for the self, but reasoning for similar and dissimilar peers did not differ. In Study 2, scores showed a step-wise increase from self, familiar peer, to unfamiliar peer, but only reasoning for an unfamiliar peer was significantly better more accurate than reasoning for the self. Reasoning for a familiar peer did not differ from reasoning for the self or for an unfamiliar peer. These results suggest that, like adults, children benefit from psychological distance when reasoning for others, but are less sensitive to degrees of social distance, showing no graded effects on performance in Study 1 and weak effects in Study 2. Stronger adult-like effects may only emerge with increasing age and development of other socio-cognitive skills.

## Introduction

Our ability to transcend the present to consider future events and anticipate psychological and physiological states allows us to adapt our behaviour to situations that have not yet transpired and to states that we are not currently experiencing. Such abilities are key in planning for both the short-term, such as bringing a snack for a long bike ride, and the long-term, such as refraining from buying a new car to save money for retirement. Research in the last decade suggests that the ability to reflect on the future and our future selves emerges early in development and undergoes significant changes during the preschool years [1]. By 3 years of age, children use language denoting the future and its uncertainty when describing what to bring on a trip [2], and between the ages of 3 and 5, children improve at planning, especially anticipating how to prevent potential problems or mishaps [3]. Children also become increasingly accurate in their prediction of future events [4, 5] and perform better at tasks requiring them to select appropriate items to fulfill future needs. This includes both hypothetical scenarios, such as bringing sunglasses for a visit to a desert [6], and real scenarios, such as selecting the necessary key to open a locked box containing a reward, or the items needed to play a game the next day [7, 8].

Future-oriented thinking can also pertain to situations involving strong emotional or motivational states such as hunger, thirst, or desires that influence how we reason about and make choices for the future. Younger children often have difficulty resisting the temptation of current rewards or pleasure, even in exchange for greater rewards or pleasure in the future [9, 10]. For example, laboratory-based tasks show that 3- to 5-year-olds have trouble refraining from using marbles in a small marbles game to save them for use with a larger marbles game [11, 12]. In addition, children have difficulty predicting future physiological and desire states in the presence of strong conflicting current states. In the “pretzel task,” researchers manipulated thirst by allowing children to consume pretzels, and when asked what they would prefer the next day—pretzels or water—children were more likely to choose water, even though they clearly preferred pretzels [13, 14]. Similarly, young children’s current desires may bias their predictions about their future desires. In Bélanger, Atance, Varghese, Nguyen, and Vendetti [15], when presented with paired child and adult items (e.g., Kool-Aid and coffee), younger preschoolers tend to choose the child items when asked what they will like when they are “all grown up,” whereas older preschoolers tend to choose the adult ones.

Although younger children typically perform poorly on these types of future-thinking tasks, interestingly, they are better able to reason about *another person’s* future. In the study described above, children correctly chose the adult items for a same-aged peer in the future more often than they did for themselves in the future [15]. Similarly, in another study [7], children more often selected the correct item to play a game in the future when choosing for another child, successfully ignoring other attractive non-functional items. Such effects have also been observed in delay of gratification tasks, where kindergarten children did not delay themselves, yet reported that another child who was “smart” would delay [16], and 3-year-olds were more likely to choose delayed rewards for the experimenter, while choosing immediate rewards for themselves [17].

### Psychological Distance

One interpretation of this beneficial “other” effect is that adopting another person’s perspective provides psychological distance from the self, here and now, and hence, from one’s current salient desires and physiological states that may bias decision-making, especially when they conflict with future goals or states. Psychological distance involves mentally separating oneself and stepping back from the immediate situation and environment. It allows greater flexibility and control in our thinking and behavior, greater reflection that is not stimulus-bound, and greater consideration of decontextualized, abstract, and goal-oriented features [18–20].

According to Trope and Liberman [20], the social dimension is one of several dimensions, in addition to spatial, temporal, and hypothetical, along which psychological distance can be conceptualized. Processing related to another person is psychologically distant from processing related to the self. Adults reason more wisely and think more creatively when solving problems for other people compared to themselves [21–23]. They also reason more wisely for *themselves* and are less emotional when instructed to adopt a distanced perspective or to treat themselves like a third person [21, 24]. Such self-distancing techniques have been used with school-aged children [25], adolescents [26], and even preschoolers [27].

However, if social distance is the critical element providing psychological distance, which in turn results in children’s improved performance when reasoning for another person, we would expect that reasoning for another person who is not socially distant would confer less, or no, benefit. That is, individuals can vary in how distant they are from the self. Liberman and Trope [28] describe four ways in which social distance can be operationalized. (1) Foremost, another person is distant from the self. However, (2) a dissimilar person is more distant than a similar person, (3) an unfamiliar person is more distant that a familiar person, and (4) a person who is part of the outgroup is more distant than a person who is part of the ingroup (see also [29, 30]).

This suggests that reasoning for another person who is familiar, similar, or part of one’s ingroup may provide less psychological distance and result in reasoning that more closely resembles reasoning for the self. When adults are asked to make intertemporal delay discounting choices for acquaintances, the more familiar and similar they rate the acquaintance, the less likely they are to make adaptive choices for them [31]. Similarly, adults generate the most creative ideas for distant others, less creative ideas for close others, and the least creative ideas for themselves [23]. Indeed, various studies have shown that performing tasks that involve processing for a familiar or similar person activates areas of the brain that overlap with those implicated in processing for the self, including regions of the ventral medial prefrontal cortex [32–34]. Moreover, such activity in these regions is sensitive to the degree of similarity, with greater perceived similarity to the self associated with greater activity [35].

In previous research where the “other” effect was observed in young children, the other person was typically a socially distant individual, such as an unfamiliar child, a child in a different kindergarten class, or an adult experimenter. In these cases, the targets of children’s reasoning were not familiar individuals, nor individuals who were particularly similar to them. In fact, little information was given about the individuals. We propose that children did well in these scenarios because of psychological distance gained from reasoning for a socially distant individual, distant not only as an individual separate from the self, but also distant on the dimensions of familiarity and similarity. If children had been asked to reason for a familiar or similar individual, and if this is akin to reasoning for oneself, then psychological distance should have been eliminated or reduced. In sum, we are interested in whether children’s reasoning can be shown to vary depending on the social distance of the other person, as has been demonstrated in adults.

### The Current Studies

In the current studies we examined whether performance on a future thinking task would differ depending on who children were asked to reason for and, more importantly, the social closeness of that person. We adopted the paradigm used by Bélanger et al. [15] to assess children’s understanding that future preferences can differ from current preferences. We tested only 3-year-olds and 4-year-olds as Bélanger et al. showed that 5-year-olds performed close to ceiling in all conditions, including reasoning about the future self. The first goal was to replicate the findings of Bélanger et al., specifically that preschoolers reason more accurately about a peer’s future preferences than about their own future preferences. The second goal was to examine whether varying the degree of social distance between the child participant and his/her peer would lead to a change in performance. Specifically, we hypothesized that children would perform best for a socially distant peer, less well for a socially close peer, and worst for themselves.

In Bélanger et al. [15], children were asked to answer for an unfamiliar child of the same age and gender. In Study 1, we created social closeness by manipulating similarity, asking children to answer for an unfamiliar child who was either similar or dissimilar to them. In Study 2, rather than create social closeness through similarity, we asked children to answer either for a familiar friend, or for an unfamiliar child. Past research has shown that preschoolers are able to differentiate friends and non-friends and tend to share more with friends [36–38], and work for a friend’s benefit [39]. Across the two studies, we tested two different dimensions of social distance (i.e., similarity and familiarity) and their effect on future thinking in young children.

## Study 1 Materials and Methods

Children were shown a series of child and adult items and asked to select which items they would like best when grown up. Children were asked to answer either for themselves, for a child who was similar to them, or for a child who was dissimilar to them. The design and methods of this study were approved by the Social Sciences and Humanities Research Ethics Board at the University of Ottawa.

### Participants

English-speaking children were recruited from a mid-sized city using a database of families who had previously expressed interest in research. Families were invited to join the research database through posters, pamphlets, children’s events, and online media. The final sample consisted of 49 children, including 24 three-year-olds (*M* = 43.58 months, *SD* = 2.80, 12 females) and 25 four-year-olds (*M* = 53.82 months, *SD* = 3.36, 14 females). Demographic information was collected from all families. A total of 83.7% of children were identified as White/Caucasian and 12.2% as mixed ethnicity (4.1% provided no information). For highest education attained, 83.7% of mothers and 77.5% of fathers reported attaining a university degree or higher. The total household income was over $80 000 for 87.8% of families.

### Procedure

Families were invited to visit the laboratory at the university campus where children were tested individually in a small quiet room. Written informed consent was obtained from parents, and oral consent was obtained from children. Parents completed a basic demographics questionnaire (e.g., ethnicity, language, income, education). Children were randomly assigned to one of three conditions: *self-future*, *similar peer-future*, and *dissimilar peer-future*. The *self-future* condition included 8 three-year-olds (4 females) and 8 four-year-olds (5 females). The *similar peer-future* condition included 8 three-year-olds (4 females) and 9 four-year-olds (5 females). The *dissimilar peer-future* condition included 8 three-year-olds (4 females), and 8 four-year-olds (4 females).

At the beginning of the session, children in both *peer-future* conditions were shown a photograph of another child who was described as either being similar (*similar peer-future)* or dissimilar (*dissimilar peer-future)* to them. To equate conditions, children in the *self-future* condition were also shown a photograph of either a similar or dissimilar child (counterbalanced), but then answered for themselves during the future preferences task. In the future preferences task, children were first administered *future* trials, in which they were shown a series of child and adult items and asked to choose which ones they would like best when they were grown up (*self-future*), or which ones the other child would like best when he/she was grown up (*peer-future* conditions). After a 1-min stretching break, children were administered *now* trials, where they were shown the same series of child and adult items, but were asked to choose which ones they or the other child liked best right now. The *now* trials served as a baseline, or “check”, that children did indeed currently prefer the child items or reasoned that another child would currently prefer child items.

After the future preferences task, several manipulation checks were administered to assess the extent to which children perceived the other child as similar or dissimilar to themselves (e.g., liking the same or different things), and whether they would behave altruistically toward a similar child and treat him/her as a friend (e.g., play and share with them). Children in the *self-future* condition also received the manipulation checks since they had been introduced to Sally/Billy.

### Similarity Manipulation

At the start of the experimental session (and prior to administering the *future* and *now* trials), the experimenter confirmed with children their birthday and which city they lived in, and asked children to name their favourite colour, food, drink and toy. Children were then shown a photograph of a child, matched for gender (“Sally/Billy”). In the *similar peer-future* condition, children were told that Sally/Billy was the same age, lived in the same city, had the same birthday, had the same favourite colour, food, drink, and toy, and was “a lot like” them. In the *dissimilar peer-future* condition, children were told that Sally/Billy was one year older, lived in a different city, had a different birthday, had a different favourite colour, food, drink, and toy, and was “not a lot like” them.

### Future Preferences Task

The protocol was closely adapted from Bélanger et al. [15]. First, children were asked to choose items that they or another child would like best in the future (5 *future* trials); then children were asked to choose items they or another child would like best right now (5 *now* trials). For the *future* trials, children were first shown a photograph of themselves taken with an instant camera (*self-future*) or a photo of Sally/Billy (*peer-future* conditions). They were then introduced to a photograph of an unfamiliar adult matched for gender. Children in the *self-future* condition were told: “Here is a picture of you. Here is a picture of Jane/John. Jane/John is a grown-up woman/man. She/he is as big as your mommy/daddy. One day you’re going to be all grown up. You’ll be as big as Jane/John. I’m going to show you some things and I want you to tell me the things you will like best when you’re all grown up.” In the *peer-future* conditions, the script was adjusted to refer to Sally/Billy.

The photographs were removed and children were presented with 5 trials of paired child and adult items, including drinks (Kool-Aid and cups of coffee), reading material (picture books and newspapers), leisure activities (sticker books and magazines), games (Play-Doh and crossword puzzles), and television shows (Dora the Explorer videos and cooking show videos). For each trial, children were shown two identical exemplars of the child item and two identical exemplars of the adult item, and asked to choose which one they or Sally/Billy would like best when grown up (e.g., “Which one of these will you like best when you’re all grown up, one of these Kool-Aid drinks, or one of these cups of coffee?”). Two fixed random orders were created for the 5 trials, with the side and order of presentation of child/adult items also randomized. The two orders were counterbalanced for *future* trials and *now* trials.

After a 1-min stretching break (e.g., reaching arms up, touching toes), children were again shown the photograph of themselves or Sally/Billy. Children in the *self-future* condition were told: “Here is a picture of you. Remember you’re ___ years old right now. I’m going to show you some things again and I want you to tell me the things that you like best right now.” In the *peer-future* conditions, the script was adjusted accordingly to refer to Sally/Billy. The photographs were removed, and in the *now* trials, children were shown the same 5 paired child and adult items and asked to choose which one they or Sally/Billy liked best right now (e.g., “Which one of these do you/Sally/Billy like best right now, one of these Kool-Aid drinks, or one of these cups of coffee?”).

A proportion score for the future preferences task was obtained by dividing the number of correct *future* trials (adult items chosen) by the number of correct *now* trials (child items chosen), yielding a score ranging from 0 to 1. However, any given *future* trial was only included in the scoring if children selected the child item on the corresponding *now* trial. Otherwise, it was impossible to know whether children were indeed adopting the perspective of their future self or another child’s future self, or simply stating their own current preference or another child’s current preference (e.g., child may answer “crossword puzzles” for a *future* trial, but is not thinking about the future and rather stating his or her current, albeit uncommon, preference). Two 4-year-old males did not select any child items on all 5 *now* trials and were excluded from the analyses.

### Similarity Manipulation Checks

After completing the future preferences task, all children were (1) shown the photo of Sally/Billy and asked if Sally/Billy was “a lot like” them or “not a lot like” them, and (2) told that Sally/Billy would be coming to the lab and asked, “Would you like to play with Sally/Billy?” (3) Then, children were presented with 4 pairs of child items (ball and bottle of bubbles, crayons and paint set, dinosaur toy and frog toy, shaker and tambourine) in a manner similar to that used in the future preferences task to determine whether children thought that Sally/Billy would like the same things as they did. For each trial, children were asked to choose which one they liked best right now, and then immediately asked to choose which one Sally/Billy liked best right now. The number of trials on which children chose the same items for themselves and Sally/Billy was recorded. (4) Finally, children were presented with 12 stickers and asked to distribute the stickers one at a time between two containers, counterbalanced for side–one with the child’s photograph, and one with Sally’s/Billy’s photograph. The number of stickers distributed to Sally/Billy was recorded.

## Study 1 Results

### Descriptive

An alpha level of .05 was used for all analyses. There was no difference in the mean age across conditions for 3-year-olds and for 4-year-olds, nor in the proportion of males and females across conditions. An analysis of variance (ANOVA) showed no significant main effect of age on future preferences scores, *F*(1, 43) = 2.42, *p* = .128, although 4-year-olds (*M* = .58, *SD* = .45) performed better than 3-year-olds (*M* = .40, *SD* = .43). The interaction between condition and age was not significant, *F*(2, 43) = 0.16, *p* = .856. Since our primary interest was to examine condition differences with respect to social distance, and Bélanger et al. [15] also did not report any interaction between condition and age, data were collapsed across the two age groups for the remainder of the analyses. Furthermore, there was no main effect of gender and no interaction between gender and condition on future preferences scores, and data were also collapsed across gender for subsequent analyses.

### Future Preferences Task

**Fig 1:**
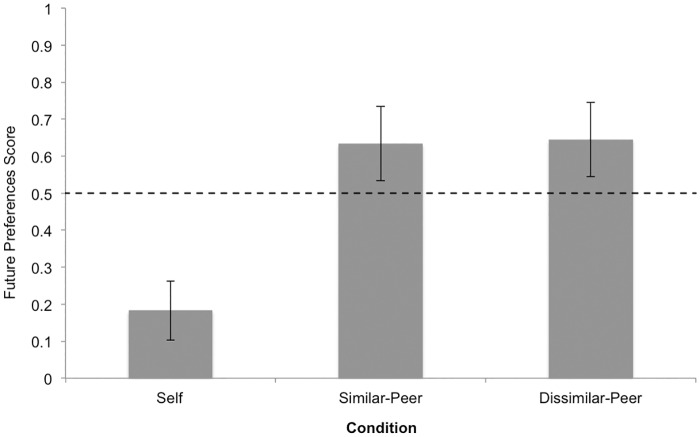
Study 1 mean future preferences scores by condition. Error bars represent standard errors of the means. Reference line refers to chance responding (i.e., score of 0.5).

A one-way ANOVA was conducted examining the effect of condition on future preferences scores. There was a significant main effect of condition, *F*(2, 46) = 7.14, *p* = .002. = *ƞ*^*2*^ = .237. Tukey post-hoc tests revealed that children in the *self-future* condition (*M* = .18, *SD* = .34) performed significantly worse than children in the *similar peer-future* condition (*M* = .63, *SD* = .42), *p* = .006, *d* = 1.14 and also significantly worse than children in the *dissimilar peer-future* condition (*M* = .64, *SD* = .42), *p* = .005, *d* = 1.16 (Fig 1). However, there was no significant difference between the two *peer-future* conditions, *p* = .996.

### Chance Analyses

We also conducted chance analyses separately for each condition to determine whether children’s performance was above, below, or no different than chance. In the *self*-*future* condition, children performed significantly below chance, *t*(15) = -3.75, *p* = .002. In the *similar peer-future* condition, children performed no different than chance, *t*(16) = 1.31, *p* = .209, and in the *dissimilar peer-future* condition, children also performed no different than chance, *t*(15) = 1.37, *p* = .191. We collapsed the two *peer-future* conditions, as they did not differ from one another, but were significantly different from the *self-future* condition, and children performed marginally above chance in the combined *peer-future* conditions (*M* = .64, *SD* = .42), *t*(32) = 1.92, *p* = .064.

### Manipulation Checks

Although children in the *self-future* condition were also administered manipulation checks, only data from children in the *peer-future* conditions are presented here since the effectiveness of our manipulation is only relevant to their performance. However, the results are similar when all available data are included. There were also some missing data from children who did not give clear answers to questions. (1) A total of 11 out of 16 (68.8%) children who were introduced to a similar child answered that Sally/Billy was “a lot like” them compared to 0 of the 16 children who were introduced to a dissimilar child, χ^2^(1, *N* = 32) = 16.76 *p* < .001, *ɸ* = .737. (2) However, there was no difference in the percentage of children choosing to play with Sally/Billy, with 14 of 17 (82.4%) of children introduced to a similar child saying “yes” and 10 of 16 (62.5%) introduced to a dissimilar child saying “yes”, χ^2^(1, *N* = 33) = 1.64, *p* = .188. (3) There was a trend showing that children introduced to a similar child were more likely to choose the same items for themselves and for Sally/Billy across the four trials (*M* = 1.94, *SD* = 1.39) compared to those introduced to a dissimilar child (*M* = 1.20, *SD* = 1.32), although this difference was not significant, *t*(30) = 1.54, *p* = .134, *d* = .428. (4) Children introduced to a similar child shared more stickers with him/her (*M* = 5.59, *SD* = 1.37), than those introduced to a dissimilar child (*M* = 3.13, *SD* = 2.80), *t*(31) = 3.24, *p* = .003, *d* = .592.

## Study 1 Discussion

We examined the effects of social distance on children’s performance on a future thinking task by comparing children’s reasoning about their own future preferences, the future preferences of a socially close other, and the future preferences of a socially distant other. We predicted that children would reason best for a child who was dissimilar (socially distant), less well for a child who was similar (socially close), and worst for themselves. We replicated the results of Bélanger et al. [15] by showing that children had more difficulty reasoning about their own future preferences compared to those of another child. When asked about their own adult preferences, children often defaulted to naming their current child preferences, such as Kool-Aid or Play-Doh, performing significantly below chance. In other words, children appeared to be biased by their current preferences, thinking that their future selves would have similar preferences. However, when asked about the adult preferences of another child, children were able to name more adult items such as cups of coffee or crossword puzzles, performing marginally above chance. Thus, when answering for another child, children did not exhibit the same “present bias” as they did when answering for themselves.

We hypothesized that children are better able to reason for another child’s future because it provides psychological distance from children’s own current desires, and thus, with less distance, we should see less benefit. Nonetheless, we failed to find any differences between reasoning for a similar child and a dissimilar child. Analyses of the manipulation checks suggest that children perceived Sally/Billy as similar or dissimilar depending on the condition. Indeed, children confirmed the similarity/dissimilarity verbally, and there was a trend towards choosing the same items more often for similar Sally/Billy than for dissimilar Sally/Billy. Furthermore, children shared more stickers with Sally/Billy in the similar condition compared to the dissimilar condition. While there was no difference in children’s willingness to play with a similar/dissimilar child, this may be related to socials norms and expectations of playing with and being nice to others regardless of similarity.

In the adult literature, similarity has been used successfully to make processing for another person closer to the processing we typically observe for the self [29, 32]. Even having the same birthday or birthday month can be sufficient to generate social closeness [23, 40–41]. Although, we too emphasized children’s favourite things, birthdays, and home city, it may be that children do not identify strongly with similarity in favourite foods, drinks, toys, and colours, since these kinds of preferences can change on a whim, as often noted by parents. Alternatively, using similarity may not be an appropriate method for stimulating closeness in young children. Although children shared more with those who they thought were similar, children may not have felt enough closeness with the other child to affect performance. Therefore, in Study 2, rather than attempting to create closeness through similarity, we capitalized on naturally occurring closeness in children’s relationships by asking children to reason for a friend.

## Study 2 Materials and Methods

Children were shown a series of child and adult items and asked to select which items they would like best when grown up. Children were asked to answer either for themselves, a familiar friend, or an unfamiliar child. As in Study 1, the design and methods of this study were approved by the same research ethics board at the University of Ottawa.

### Participants

English-speaking children were recruited from the same city using primarily the same methods as those described in Study 1. However, a small number of children were tested offsite at a local daycare centre (*n* = 11), and at an aviation and space museum (*n* = 22). Analyses revealed no significant differences in children’s performance across these three locations. Families at the daycare received information and consent forms from the daycare director and only those children whose parents returned signed consent forms were tested. Families at the museum were approached during their visit and invited to participate in a short study onsite. Due to time constraints, children recruited and tested at the museum received a shortened protocol that included only the first manipulation check (i.e., asking about peer’s similarity/dissimilarity) instead of all four checks.

The final sample consisted of 81 children, including 42 three-year-olds (*M* = 43.17 months, *SD* = 2.88, 23 females), and 39 four-year-olds (*M* = 54.44 months, *SD* = 2.90, 20 females). Two children, one 3-year-old male and one 3-year-old female, were excluded because they did not select any child items during the *now* trials. Demographic information was collected from all families, except those tested at the daycare. A total of 55.6% of children were identified as White/Caucasian, 17.3% as mixed ethnicity, and 2.5% as Asian (24.6% provided no information). For highest education attained, 64.2% of mothers and 70.5% of fathers reported attaining a university degree or higher (14.8% provided no information). The total household income was over $80 000 for 70.4% of families (17.3% provided no information).

### Procedure

All children were tested individually in a small quiet room. Written informed consent was obtained from parents, and oral consent was obtained from children. Parents at the lab and at the museum completed the same demographics questionnaire as in Study 1. Children were randomly assigned to one of three conditions: *self-future*, *friend-future*, and *non-friend-future*. The *self-future* condition included 14 three-year-olds (8 females) and 13 four-year-olds (7 females). The *friend-future* condition included 17 three-year-olds (9 females) and 13 four-year-olds (7 females). The *non-friend-future* condition included 11 three-year-olds (6 females), and 13 four-year-olds (6 females). In the waiting room, in the *self-future* and *friend-future* conditions, children were asked about a best friend that they like to play with, along with the friend’s age. Parents confirmed the appropriateness of the named friend (e.g., not an adult or imaginary friend) in all cases, except for 7 children tested at the daycare where parents were not present. Nine children named a sibling and were unable to name a friend when prompted further, so the sibling was used in the procedures below. However, only 2 of them were in the *friend-future* condition.

At the beginning of the session, all children were asked to draw a picture of themselves with guidance from the experimenter. Children were provided with a small piece of paper with a template of a face (e.g., black circle outline for face), and several coloured crayons. Children’s names were written at the top of the paper. Children were then asked to draw a picture of either a friend (*self-future* and *friend-future* conditions) or a picture of an unfamiliar child, matched for age and sex, named “Sally/Billy” (*non-friend-future* condition). Although children in the *self-future* condition were also asked to draw a friend to equate the conditions as much as possible, they nonetheless answered for themselves in the future preferences task. Children in the *non-friend-future* condition were shown a photograph of Sally/Billy (“This is Sally/Billy”) and then asked to draw a picture of Sally/Billy. The name of the friend or Sally/Billy was written at the top of the paper and the photograph of Sally/Billy was removed. After both drawings were completed, the experimenter asked children to identify each drawing, and any errors were corrected. Drawings were used instead of photographs due to ethical and practical concerns with obtaining and using photographs of children’s friends. A similar procedure has been used previously when children were asked to allocate resources between themselves, a classmate friend, a classmate non-friend, and an unfamiliar child [37].

Children then completed the future preferences task following the same procedures used in Study 1. Children completed 5 *future* trials, a 1-min stretching break, followed by 5 *now* trials. After the future preferences task, we administered the same manipulation checks used in Study 1. We did this to obtain a baseline measure of how children perceive the similarity of an unfamiliar child when given no additional information, and the extent to which they would behave altruistically toward the child. Importantly, we were interested in how this compares to the similarity that children perceive for a friend and whether they would behave more altruistically toward a friend, as suggested by past research [36–37]. In doing so, we hoped to gain insight about how similarity may or may not affect closeness in young children, and thus allow us to further interpret our Study 1 results.

### Future Preferences Task

The same procedures described in Study 1 were used, although drawings were used instead of photographs.

### Similarity Manipulation Checks

The same four manipulation checks described in Study 1 were administered to children.

## Study 2 Results

### Descriptive

An alpha level of .05 was used for all analyses. There was no difference in the mean age across conditions for 3-year-olds and 4-year-olds, nor in the proportion of males and females across conditions. There was a marginally significant main effect of age, *F*(1, 75) = 3.09, *p* = .083, with 4-year-olds (*M* = .43, *SD* = .42) performing better than 3-year-olds (*M* = .27, *SD* = .36). However, there was no interaction between age and condition, *F*(2, 75) = .485, *p* = .618 and, as in Study 1, data were collapsed across age groups for the remainder of the analyses. Furthermore, there was no main effect of gender and no interaction between gender and condition on future preferences scores, and data were also collapsed across gender for subsequent analyses.

### Future Preferences Task

**Fig 2:**
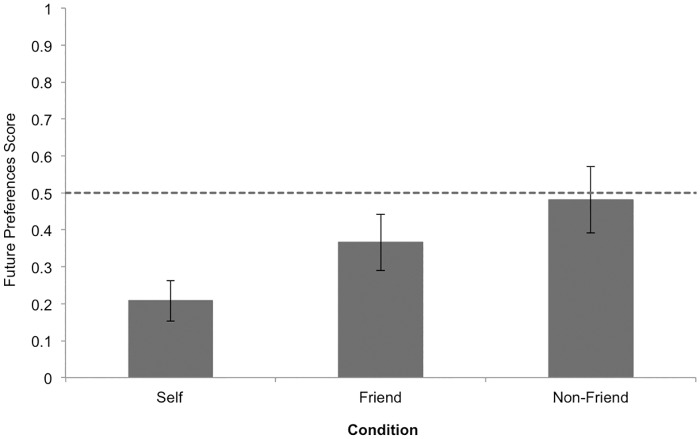
Study 2 mean future preferences scores by condition. Error bars represent standard errors of the means. Reference line refers to chance responding (i.e., score of 0.5).

A one-way ANOVA was conducted examining the effect of condition on future preferences scores. The main effect of condition was significant, *F*(2, 78) = 3.23, *p* = .045, *ƞ*^*2*^ = .077 and Tukey post-hoc tests revealed that children in the *non-friend-future* condition (*M* = .48, *SD* = .44) performed significantly better than children in the *self-future* condition (*M* = .21, *SD* = .29), *p* = .036, *d* = .708 (Fig 2). However, children in the *friend-future* condition (*M* = .37, *SD* = .42) did not differ from children in the *self-future* condition, *p* = .278, nor from children in the *non-friend-future* condition, *p* = .523.

### Chance Analyses

We also conducted analyses separately for each condition to determine whether children’s performance was above, below, or no different than chance. Children in the *self*-*future* condition performed significantly below chance, *t*(26) = -5.28, *p* < .001, whereas children in the *friend-future* condition preformed marginally below chance, *t*(29) = -1.76, *p* = .089, and children in the *non-friend-future* condition performed no different than chance, *t*(23) = -2.09, *p* = .836.

### Manipulation Checks

Although children in the *self-future* condition were also administered manipulation checks, only data from children in the *friend- and non-friend-future* conditions are presented. However, the results are similar when all available data are included. As noted above, children tested at the museum only completed the first manipulation check. Furthermore, there were some missing data from children who did not give clear answers to questions. (1) There was no significant difference in how often children answered that their friend was “a lot like” them, 15 out of 29 (51.7%), and how often children answered that Sally/Billy was “a lot like” them, 14 out of 19 (73.7%), χ^2^(1, *N* = 48) = 2.32 *p* = .111. (2) There was no difference in the percentage of children choosing to play with their friend, 25 out of 26 (96.2%), and those choosing to play with Sally/Billy, 13 of 15 (86.7%), χ^2^(1, *N* = 41) = 1.26, *p* = .299. (3) There was no difference in the number of same items children picked for their friend (*M* = 1.85 *SD* = 1.43) and for Sally/Billy (*M* = 1.46, *SD* = 1.51, *t*(38) = -0.79, *p* = .432. (4) Surprisingly, there was also no difference in the number of stickers children shared with their friend (*M* = 4.96, *SD* = 2.38) and with Sally/Billy (*M* = 5.13, *SD* = 2.09), *t*(41) = 0.22, *p* = .823.

## Study 2 Discussion

In Study 1 we examined the effects of social distance on children’s performance on a future thinking task by comparing children’s reasoning about their own future preferences, the future preferences of a similar child, and the future preferences of a dissimilar child. Whereas in Study 1, we manipulated social distance by varying similarity, in Study 2 we manipulated social distance by varying familiarity. We hypothesized that children would reason best for an unfamiliar non-friend (socially distant), less well for a familiar friend (socially close), and worst for themselves.

We found that children reasoned correctly on more trials for a non-friend than for themselves, replicating the results of Study 1 and Bélanger et al. [15], and again demonstrating the advantage in reasoning for another person. As in Study 1, when reasoning for themselves, children appeared to be systematically biased by their current preferences, but when reasoning for a non-friend, children did not show this same bias. Nonetheless, when answering for a non-friend, children performed only at chance, suggesting that while they no longer were systematically biased by their current preferences, they were also not yet consistently able to answer that a peer would prefer an adult item in the future. More data with a broader age range is needed to better understand whether this kind of reasoning reflects a transitional stage in children’s reasoning. For example, it may be that, at a younger age (e.g., older 2-year-olds), children are systematically biased by their current preferences even when reasoning about another child’s future preferences, then begin to appreciate that future preferences may differ from current ones (though still responding at chance) and, finally, gain a full appreciation of this fact as reflected in above-chance performance.

Unlike the non-friend condition, children’s performance in the friend condition did not differ from performance in the self condition nor did it differ from performance in the non-friend condition, despite mean scores showing a step-wise increase across the groups. When compared with data from Study 1, performance in the friend condition was significantly lower than performance in the similar/dissimilar peer conditions, *F*(1, 61) = 6.76, *p* = .012, *ƞ*^*2*^ = .100, while the self conditions across the studies did not differ *F*(1, 41) = 0.07, *p* = .797, nor did the non-friend and similar/dissimilar peer conditions, *F*(1, 55) = 1.91, *p* = .173. Overall, these findings offer some preliminary support for our hypothesis that reasoning for a socially close friend differs from reasoning for an unfamiliar peer, although effects are small.

There were no differences observed in any of the manipulation checks when comparing children in the friend and non-friend conditions. However, interesting patterns emerged when comparing the results to those of Study 1. Over 70% of children answered that Sally/Billy was a lot like them even though they were given little information about Sally/Billy in Study 2, a rate comparable to that observed for a similar peer in Study 1 (68.8%). It may be that, by default, children assume that an unfamiliar peer is more similar than dissimilar to them. In contrast, only about 50% of children reported their friend as being a lot like them, possibly reflecting actual knowledge of their friends–that friends can be both similar and dissimilar to oneself. As in Study 1, a high percentage of children in both conditions reported wanting to play with Sally/Billy, with the highest percentage across both studies reported for friends, and the lowest for a dissimilar peer. The number of same items children picked for friends was similar to that picked for a similar peer in Study 1, whereas the lowest number of same items picked was for a dissimilar peer in Study 1. Surprisingly, children did not share more stickers with their friends, contrary to previous research [36–38]. Indeed, the mean number of stickers shared was similar across friends, non-friends, and similar peers. Only for dissimilar peers did children choose to share less stickers. These patterns suggest that children may initially assume unfamiliar peers are similar to themselves, but after having actual experience with peers who then become friends, they no longer make that assumption. Furthermore, unfamiliar peers are treated altruistically as are friends, with children sharing just under half their stickers with them. Interestingly, children tend to treat dissimilar peers differently than other peers.

Despite our findings that friends were not treated as particularly different from other peers, children seemed to reason differently for friends on the future preferences task. Children appeared to be more biased towards present preferences when reasoning for their friends, performing marginally worse than chance. When reasoning for non-friends, however, children did not show this same bias, although their performance was still at chance level. These results are consistent with our hypothesis, although stronger evidence in the future would distinguish three distinct levels of performance.

## General Discussion

Although young children often have difficulty thinking about the future—whether it be planning for future needs, delaying immediate gratification for future rewards, or understanding that desires and states can change with time—there is an interesting divergence in performance between self and other. More specifically, when given the opportunity to reason for another person, children often perform more accurately, reason more adaptively, and are less likely to be biased by the present than when asked to reason about themselves. This advantage in performance is consistent with the adult literature showing that thinking for another person, or cognitively adopting a third person perspective when thinking for oneself, provides psychological distance from the task and emotions at hand. This in turn is associated with greater wisdom, more adaptive decision-making, and better emotional regulation [21, 24, 31]. In addition, both behavioural and neuropsychological studies have shown that reasoning for a close other resembles reasoning for the self, and confers less benefits than reasoning for somebody who is distant from self [23, 31, 35].

In our studies, we were interested in whether an effect of social distance could be demonstrated in children using Bélanger et al.’s [15] future preferences task. Specifically, we manipulated the social distance of the peer that children were asked to reason for, varying the similarity of the peer in Study 1, and varying the familiarity of the peer in Study 2. Across both studies, we hypothesized that children would reason best for a socially distant peer, less well for a socially close peer, and worst for themselves. We replicated the effect reported in Bélanger et al. that children were less likely to be biased by the present and were more likely to chose adult items when reasoning about a peer’s future preferences than when reasoning about their own future preferences. This was true for both similar and dissimilar peers in Study 1 and for non-familiar peers in Study 2, showing a robust effect.

Nonetheless, we did not find any graded effects of performance by varying social distance in Study 1. Children did not reason differently for similar (i.e., socially closer) peers. In fact, performance was the same as that for dissimilar peers, and both were more accurate than performance for self. Since similarity may not engender closeness in young children as it does in adults [23, 32], in Study 2, we focused on preexisting closeness in children’s friendships. Although, here, we found no clear difference between children’s reasoning for friends and non-friends, there was also no clear difference between children’s reasoning for friends and for themselves. The mean performance for friends fell in between that for self and for non-friends. These results suggest that children may have reasoned differently for their friends, and that perhaps there is greater social distance between a friend and non-friend than between a similar and dissimilar peer. However, it appears that the effects were small and less differentiated than those seen in adults.

Younger children may be able to broadly distinguish between “self” and “other” in their thinking, yet cannot readily differentiate “other” further. That is, their thinking and behaviour may be less sensitive to the social category or status of others. For example, 3- and 4-year olds do not consistently favour the ingroup over the outgroup when sharing their own resources or when directing the distribution of resources by others [42–43]. Furthermore, while studies have shown that children are more willing to share with and work for friends [37–39], participants were often older preschoolers and children, and at least one study reported that only 4-year-olds, and not 3-year-olds, showed a preference for friends in sharing behaviour [37]. However, in both Studies 1 and 2, we did not observe such interactions between sharing behaviour and age.

Nonetheless, children in Study 1 did share more with similar than dissimilar peers, but there were no associated differences seen in performance across the two conditions. Perhaps similarity is not strong enough to invoke feelings of closeness to reduce psychological distance between the self and other. In Study 2, children did not differentiate friends and non-friends in their sharing behaviours, nor in how likely they were to hold the same item preferences, but, interestingly, seemed to reason differently for their friends’ future preferences. This is in contrast to the pattern of behaviour seen in Study 1. Together, these findings suggest that behaviours such as sharing and having the same preferences may not be good indicators of how close children feel to another person and how it may affect their thinking. Relatedly, the ability to distinguish socially close and socially distant others may not necessarily imply that young children will also have qualitatively different experiences when adopting the perspectives of these respective individuals. In other words, the capacity to make distinctions along the dimension of social distance may be necessary but not sufficient for the effects of psychological distance to then be detected in children’s future-oriented reasoning.

Children’s understanding of their own and others’ mental states including beliefs, thoughts, and emotions, develops significantly during the preschool years [44]. Also known as theory of mind, this understanding facilitates the ability to mentally distance oneself from one’s own mental states to take the perspective of another person. Such theory of mind skills may be important for whether young children experience different processes when adopting the perspective of a close versus a distant other, especially when the difference is subtle, such as that arising from similarity. For example, White and Carlson [27] found that theory of mind was associated with children’s ability to benefit from psychological distancing techniques. Preschoolers were instructed to speak to themselves in the third person or to assume a character (e.g., Batman) during a cognitive flexibility task, and facilitative effects were seen only in those who performed better on theory of mind tasks. Thus, a certain level of theory of mind reasoning and perspective taking may be required before children can consistently respond to nuanced and graded differences in psychological distance. Furthermore, our task required future thinking, and it has been suggested that theory of mind may be involved in children’s ability to think about and adopt the perspective of their future self [45–47]. Including measures of theory of mind in our current studies would have potentially allowed us to better understand our findings and is an important direction for future research on this topic.

Another important addition for future research is to develop a clear measure of closeness in young children. As discussed earlier, it appears that sharing and having the same preferences are not consistent indicators of closeness in young children. Although it is not obvious what an ideal measure of closeness for young children may be, it is possible that our Study 2 would have shown more distinct group differences had we assessed or controlled for children’s actual closeness with their friends. In some cases, named friends were clearly individuals with whom the children had a strong unique relationship; in other cases, friends were simply children they played with at daycare. Children’s friendships at ages 3 and 4 can also be unstable and fleeting. Variation in relationship closeness may have diluted our effects. Accordingly, in future research, it will be important to examine stronger, more consistent relationships in children’s lives, such as sibling relationships. There may be less variation across children with respect to their closeness with a family member. Indeed, differences in children’s reasoning have been demonstrated for unfamiliar adults versus a family member. For example, Bélanger et al. [15] showed that even 3-year-olds were able to choose adult items instead of child items when asked to name the preferences of an unfamiliar adult. Yet, in a prior study by Atance, Bélanger, and Meltzoff [48], both 3- and 4-year-olds had difficulty choosing an adult item instead of a child item as a gift for their mothers.

Our findings also suggest that including 5-year-olds and perhaps even older children may be a helpful next step in this line of work, given that older children may have more stable friendships and superior theory of mind skills. Although the task we used would lead to ceiling effects in older children, a modified version of it has successfully been used with adults [49] and could also be adapted for use with older children.

To better understand the possible benefits of psychological distance on future-oriented reasoning, we attempted to decrease the accuracy of children’s predictions about another person’s future preferences by decreasing psychological distance via social distance. An alternative approach, however, is to increase performance for the self by increasing psychological distance. The recent findings from White and Carlson [27] suggest that at least older preschoolers are sensitive to cognitive distancing techniques, and similar methods could be applied to reasoning about the future. However, in White and Carlson, children were engaged in psychological distancing within an abstract card-sorting task, whereas the future preferences task relates directly to the self. This may limit the types of methods that can be used, especially those related to social distance. For example, assuming the perspective of a character, as children did in White and Carlson, fundamentally changes the future preferences task as the predictions no longer pertain to the self, but to a third person. However, it may be possible to use methods that manipulate psychological distance along other dimensions identified by Trope and Liberman [20], such as hypothetical/pretense.

When we are confronted with difficult tasks, especially those that involve strong biases or emotions, adopting the perspective of another person can lead to more adaptive decisions and behaviours. Like adults, children can benefit from the psychological distance afforded by reasoning for another person but, unlike adults, our results suggest that children are not similarly influenced by the social distance or status of the other individual, which might explain why we did not observe the same graded effects in performance that covary with social distance in adults. However, in cases where differences between individuals are greater and based on preexisting relationships, such as comparing friends and non-friends, small differences begin to emerge. With increasing age and maturing theory of mind abilities, however, such differences may become more marked and begin to mirror those observed in adults.

## Supporting Information

S1 File Lee & Atance datafile. (SAV)

## References

[1] Atance CM. Young children's thinking about the future. Child Dev. 2015;9: 178–82.

[2] Atance CM, O’Neill DK. Preschoolers’ talk about future situations. First Lang. 2005;25: 5–18.

[3] Hudson JA, Shapiro LR, Sosa BB. Planning in the real world: Preschool children's scripts and plans for familiar events. Child Dev. 1995;66: 984–98. 7671660

[4] Busby J, Suddendorf T. Recalling yesterday and predicting tomorrow. Cogn Dev. 2005; 20: 362–72.

[5] Quon E, Atance CM. A comparison of preschoolers' memory, knowledge, and anticipation of events. J Cogn Dev. 2010;11: 37–60.

[6] Atance CM, Meltzoff AN. My future self: Young children's ability to anticipate and explain future states. Cogn Dev. 2005;20: 341–61. 23956493 10.1016/j.cogdev.2005.05.001 PMC3744374

[7] Russell J, Alexis D, Clayton N. Episodic future thinking in 3- to 5-year-old children: The ability to think of what will be needed from a different point of view. Cognition. 2010;114: 56–71. 10.1016/j.cognition.2009.08.013 19781693

[8] Suddendorf T, Nielsen M, Von Gehlen R. Children’s capacity to remember a novel problem and to secure its future solution. Dev Sci. 2011;14: 26–33. 10.1111/j.1467-7687.2010.00950.x 21159085

[9] Mischel W. Processes in delay of gratification In: Berkowitz L, editor. Advances in experimental social psychology. Vol. 7 San Diego, CA: Academic Press; 1974 p. 249–92.

[10] Thompson C, Barresi J, Moore C. The development of future-oriented prudence and altruism in preschoolers. Cogn Dev. 1997;12: 199–212.

[11] Lee WS, Carlson SM. Knowing when to be “rational”: Flexible economic decision making and executive function in preschool children. Child Dev. 2015;86: 1434–48. 10.1111/cdev.12401 26264807

[12] Metcalf JL, Atance CM. Do preschoolers save to benefit their future selves? Cogn Dev. 2011;26: 371–82.

[13] Atance CM, Meltzoff AN. Preschoolers' current desires warp their choices for the future. Psychol Sci. 2006;17: 583–7. 10.1111/j.1467-9280.2006.01748.x 16866743 PMC1523428

[14] Mahy CE, Grass J, Wagner S, Kliegel M. These pretzels are going to make me thirsty tomorrow: Differential development of hot and cool episodic foresight in early childhood? Br J Dev Psychol. 2014;32: 65–77. 10.1111/bjdp.12023 24219388

[15] Bélanger MJ, Atance CM, Varghese AL, Nguyen V, Vendetti C. What will I like best when I'm all grown up? Preschoolers' understanding of future preferences. Child Dev. 2014; 85: 2419–31. 10.1111/cdev.12282 25109689

[16] Nisan M, Koriat A. Children's actual choices and their conception of the wise choice in a delay-of-gratification situation. Child Dev. 1977;48: 488–94.

[17] Prencipe A, Zelazo PD. Development of affective decision making for self and other: Evidence for the integration of first- and third-person perspectives. Psychol Sci. 2005;16: 501–5. 10.1111/j.0956-7976.2005.01564.x 16008779

[18] Carlson SM, Zelazo PD. Symbolic thought In: Haith MM, Benson JB, editors. Encyclopedia of infant and early childhood development. Vol. 3 London: Elsevier; 2008 p. 288–97.

[19] Sigel IE. The distancing hypothesis: A causal hypothesis for the acquisition of representational thought In: Jones MR, editor. Miami symposium on the prediction of behavior. Coral Gables, FL: University of Miami Press; 1968 p. 99–118.

[20] Trope Y, Liberman N. Construal-level theory of psychological distance. Psychol Rev. 2010;117: 440–63. 10.1037/a0018963 20438233 PMC3152826

[21] Grossmann I, Kross E. Exploring Solomon’s Paradox: Self-distancing eliminates the self-other asymmetry in wise reasoning about close relationships in younger and older adults. Psychol Sci. 2014;25: 1571–80. 10.1177/0956797614535400 24916084

[22] Kross E, Grossmann I. Boosting wisdom: Distance from the self enhances wise reasoning, attitudes, and behavior. J Exp Psychol Gen. 2012;141: 43–8. 10.1037/a0024158 21728454

[23] Polman E, Emich KJ. Decisions for others are more creative than decisions for the self. Pers Soc Psychol Rev. 2011;37: 492–501. 10.1177/0146167211398362 21317316

[24] Kross E, Ayduk O, Mischel W. When asking “why” does not hurt: Distinguishing rumination from reflective processing of negative emotions. Psychol Sci. 2005;16: 709–15. 10.1111/j.1467-9280.2005.01600.x 16137257

[25] Kross E, Duckworth A, Ayduk O, Tsukayama E, Mischel W. The effect of self-distancing on adaptive versus maladaptive self-reflection in children. Emotion. 2011;11: 1032–9. 10.1037/a0021787 21728415

[26] White RE, Kross E, Duckworth AL. Spontaneous self-distancing and adaptive self-reflection across adolescence. Child Dev. 2015;86: 1272–81. 25876213 10.1111/cdev.12370 PMC4607548

[27] White RE, Carlson SM. What would Batman do? Self-distancing improves executive function in young children. Dev Sci. 2016;19: 419–26. 10.1111/desc.12314 25997842

[28] Liberman N, Trope Y. Traversing psychological distance. Trends Cogn Sci. 2014;18: 364–9. 10.1016/j.tics.2014.03.001 24726527

[29] Liviatan I, Trope Y, Liberman N. Interpersonal similarity as a social distance dimension: Implications for perception of others’ actions. J Exp Soc Psychol. 2008;44: 1256–69. 10.1016/j.jesp.2008.04.007 19352440 PMC2665912

[30] Stephan E, Liberman N, Trope Y. The effects of time perspective and level of construal on social distance. J Exp Soc Psychol. 2011;47: 397–402. 10.1016/j.jesp.2010.11.001 21836728 PMC3153444

[31] Kim H, Schnall S, White MP. Similar psychological distance reduces temporal discounting. Pers Soc Psychol Rev. 2013;39: 1005–16. 10.1177/0146167213488214 23653066

[32] Mitchell JP, Macrae CN, Banaji MR. Dissociable medial prefrontal contributions to judgments of similar and dissimilar others. Neuron. 2006;50: 655–63. 10.1016/j.neuron.2006.03.040 16701214

[33] Schmitz TW, Kawahara-Baccus TN, Johnson SC. Metacognitive evaluation, self-relevance, and the right prefrontal cortex. Neuroimage. 2004;22: 941–7. 10.1016/j.neuroimage.2004.02.018 15193625

[34] Vanderwal T, Hunyadi E, Grupe DW, Connors CM, Schultz RT. Self, mother and abstract other: An fMRI study of reflective social processing. Neuroimage. 2008;41: 1437–46. 10.1016/j.neuroimage.2008.03.058 18486489 PMC2559963

[35] Mitchell JP, Banaji MR, MacRae CN. The link between social cognition and self-referential thought in the medial prefrontal cortex. J Cogn Neurosci. 2005;17: 1306–15. 10.1162/0898929055002418 16197685

[36] Garon N, Johnson B, Steeves A. Sharing with others and delaying for the future in preschoolers. Cogn Dev. 2011;26: 383–96.

[37] Moore C. Fairness in children's resource allocation depends on the recipient. Psychol Sci. 2009;20: 944–8. 10.1111/j.1467-9280.2009.02378.x 19515118

[38] Paulus M, Licata M, Kristen S, Thoermer C, Woodward A, Sodian B. Social understanding and self-regulation predict pre-schoolers’ sharing with friends and disliked peers A longitudinal study. Int J Behav Dev. 2015;39: 53–64.

[39] Kanfer FH, Stifter E, Morris SJ. Self-control and altruism: Delay of gratification for another. Child Dev. 1981;52: 674–82.

[40] Gunia BC, Sivanathan N, Galinsky AD. Vicarious entrapment: Your sunk costs, my escalation of commitment. J Exp Soc Psychol. 2009;45: 1238–44.

[41] Miller DT, Downs JS, Prentice DA. Minimal conditions for the creation of a unit relationship: The social bond between birthdaymates. Eur J Soc Psychol. 1998;28: 475–81.

[42] DeJesus JM, Rhodes M, Kinzler KD. Evaluations versus expectations: Children's divergent beliefs about resource distribution. Cogn Sci. 2014;38: 178–93. 10.1111/cogs.12093 24117730

[43] Fehr E, Bernhard H, Rockenbach B. Egalitarianism in young children. Nature. 2008;454: 1079–83. 10.1038/nature07155 18756249

[44] Wellman HM, Liu D. Scaling of theory-of-mind tasks. Child Dev. 2004;75: 523–41. 10.1111/j.1467-8624.2004.00691.x 15056204

[45] Atance CM, O’Neill DK. The emergence of episodic future thinking in humans. Learning Motiv. 2005;36: 126–44.

[46] Moore C, Barresi J, Thompson C. The cognitive basis of future-oriented prosocial behavior. Soc Dev. 1998;7: 198–218.

[47] Suddendorf T, Corballis MC. Mental time travel and the evolution of the human mind. Genet Soc Gen Psychol Monogr. 1997;123: 133–67. 9204544

[48] Atance CM, Bélanger M, Meltzoff AN. Preschoolers' understanding of others' desires: Fulfilling mine enhances my understanding of yours. Dev Psychol. 2010;46: 1505–13. 10.1037/a0020374 20677859

[49] Renoult L, Kopp L, Davidson PSR, Taler V, Atance CM. You’ll change more than I will: Adults’ predictions about their own and others’ future preferences. Q J Exp Psychol. 2016;69: 299–309. 10.1080/17470218.2015.1046463 26211536

